# Luspatercept ameliorates disease phenotype and complications in the Townes mouse model of sickle cell disease

**DOI:** 10.1172/JCI197706

**Published:** 2026-02-02

**Authors:** Maiko Sezaki, Tian Li, Mingzhe Pan, Zhihong Wang, Jie Bai, Justin G. Horowitz, Julia Z. Xu, Gang Huang

**Affiliations:** 1 Department of Cell Systems and Anatomy, UT Health San Antonio, San Antonio, Texas, USA.; 2 Department of Hematology, Fujian Provincial Hospital, Fuzhou, China.; 3 Department of Pharmacy Services, UT Health San Antonio MD Anderson Cancer Center, San Antonio, Texas, USA.; 4 Division of Hematology/Oncology, Department of Medicine, University of Pittsburgh, Pennsylvania, USA.; 5 Department of Pathology and Laboratory Medicine, UT Health San Antonio, Joe R. and Teresa Lozano Long School of Medicine, San Antonio, Texas, USA.

**Keywords:** Hematology, Immunology, Hematopoietic stem cells

## Abstract

Preclinical data demonstrating that luspatercept mitigates vaso-occlusive and anemia-related complications in the Townes sickle cell model provide a strong rationale for its evaluation in SCD patients.

**To the Editor:** The point substitution from glutamic acid to valine at codon 6 of the β-globin gene underlies sickle cell disease (SCD) pathobiology, resulting in highly adhesive sickling RBCs under hypoxic conditions that cause vaso-occlusive crisis (VOC) and hemolytic anemia. Multiorgan complications prevalent in patients with SCD are exacerbated by a chronic anemic state and contribute to heightened morbidity and mortality (1). As blood transfusions can cause unwanted complications (iron overload, hemolytic transfusion reactions) and the use of erythroid-stimulating agents (ESAs) currently lacks consensus due to limited clinical data and inconsistent results, alternative therapeutic options for treating chronic anemia have been actively sought after. Here, we present preclinical evidence that luspatercept, a TGF-β ligand–trapping fusion protein recently approved by the FDA for the treatment of β-thalassemia and low-risk myelodysplastic syndromes failing ESAs, shows favorable outcomes in the Townes SCD model and protects against vaso-occlusive and anemia-related complications.

The Townes SCD model, generated by replacing mouse α-globin with human α-globin and mouse β-globin with human sickle β- and fetal γ-globin, mimics clinically relevant features of patients with SCD (2). We initially noted that 30 mg/kg of luspatercept treatment significantly prolonged the survival of SCD mice and improved peripheral blood (PB) parameters, including RBC, hemoglobin (HGB), and hematocrit (HCT) levels, but not with erythropoietin (Figure 1, A and B, and Supplemental Figure 1, A–D; supplemental material available online with this article; https://doi.org/10.1172/JCI197706DS1). Histological examination of luspatercept-treated PB smears revealed a reduction in abnormal and sickling RBCs, and an increase in the proportion of morphologically normal cells (Figure 1, C and D). We also found a significant decline in circulating reticulocytes (Figure 1E), which are generally increased in patients with SCD. The middle fluorescent ratio (MFR) fraction of reticulocytes, evidently reduced by luspatercept treatment (Figure 1F), has also been identified as a sensitive predictor of VOC (3). A pulse-chase experiment to determine RBC turnover rate confirmed a greater percentage of fluorescence-retaining RBCs in luspatercept-treated PB (Figure 1, G and H, and Supplemental Figure 1, E and F), indicating better-quality RBCs. This could result from less mitochondrial content in mature RBCs (Figure 1, I and J, and Supplemental Figure 1G), which has recently been associated with increased sickling tendency and oxidative stress in patients with SCD (4).

To further characterize the luspatercept response in SCD mice, we next examined upstream erythroid lineages in the bone marrow (BM) and spleen. BM imaging of femurs revealed a significant decrease in immature nucleated Ter119-positive cells in luspatercept-treated mice (Supplemental Figure 2, A and B), while flow cytometric analysis was consistent in showing a marked increase only in terminal RBCs (gate V) (Figure 1, K and L). Chronic anemia eventually impacts BM-residing hematopoietic stem cells (HSCs), which are dormant and only activated out of quiescence to manage stress-induced and pathological conditions. Interestingly, we noticed a significant recovery of BM short-term HSCs and a reduction in the megakaryocyte/erythroid-biased MPP2 subset, which suggests a relief from anemia-induced stress hematopoiesis (Supplemental Figure 2, C and D). Thus, the perturbations of HSCs reported in patients with SCD (reduced BM CD34-positive cells), recapitulated in the Townes model, were partially rescued. Furthermore, extramedullary hematopoiesis (EMH) in the spleen, which usually compensates for compromised BM function, was mitigated (Figure 1, M and N). The decrease in splenic mature RBCs and upstream megakaryocyte-erythroid progenitors (MEPs) suggests less dependence on EMH (Supplemental Figure 3, A–D). This is likely supported by the marked increase in CD11b and F4/80 double-positive splenic red pulp macrophages (RPMs), resulting in efficient clearing of erythroblasts for iron recycling and hemoglobin synthesis through upregulated CD163 expression (Figure 1, O and P, and Supplemental Figure 3, E and F).

Finally, increased adherence of sickle RBCs to the vascular endothelium has been reported to initiate VOC, which can be scavenged by circulating patrolling monocytes (pMos) (5). We further demonstrate that despite lowering the CD11b and CD115 double-positive population, which includes all monocytes and macrophages, including those that can cause VOC, the ratio of PB patrolling-to-classical monocytes (cMos) is increased with luspatercept treatment (Figure 1, Q–S).

Managing chronic anemia is a precarious balancing act in SCD, whereby simply increasing the number of unhealthy sickle RBCs could also raise blood viscosity and counterintuitively promote vaso-occlusion. In summary, we provide preclinical evidence of the efficacy of luspatercept in SCD on multiple fronts, but not via fetal hemoglobin (Supplemental Figure 3, G and H). These results provide a strong rationale for conducting clinical trials to investigate the efficacy of luspatercept in patients with SCD.

## Funding support

This work is the result of NIH funding, in whole or in part, and is subject to the NIH Public Access Policy. Through acceptance of this federal funding, the NIH has been given the right to make the work publicly available in PubMed Central.

- NIH grants R01 CA248019 and R01 CA266256 (to GH).
- Department of Defense grant W81XWH2110148 (to GH).
- NIH Cancer Center Support Grant P30 CA054174 (to GH).

## Supplementary Material

Supplemental data

Supporting data values

## Figures and Tables

**Figure 1:**
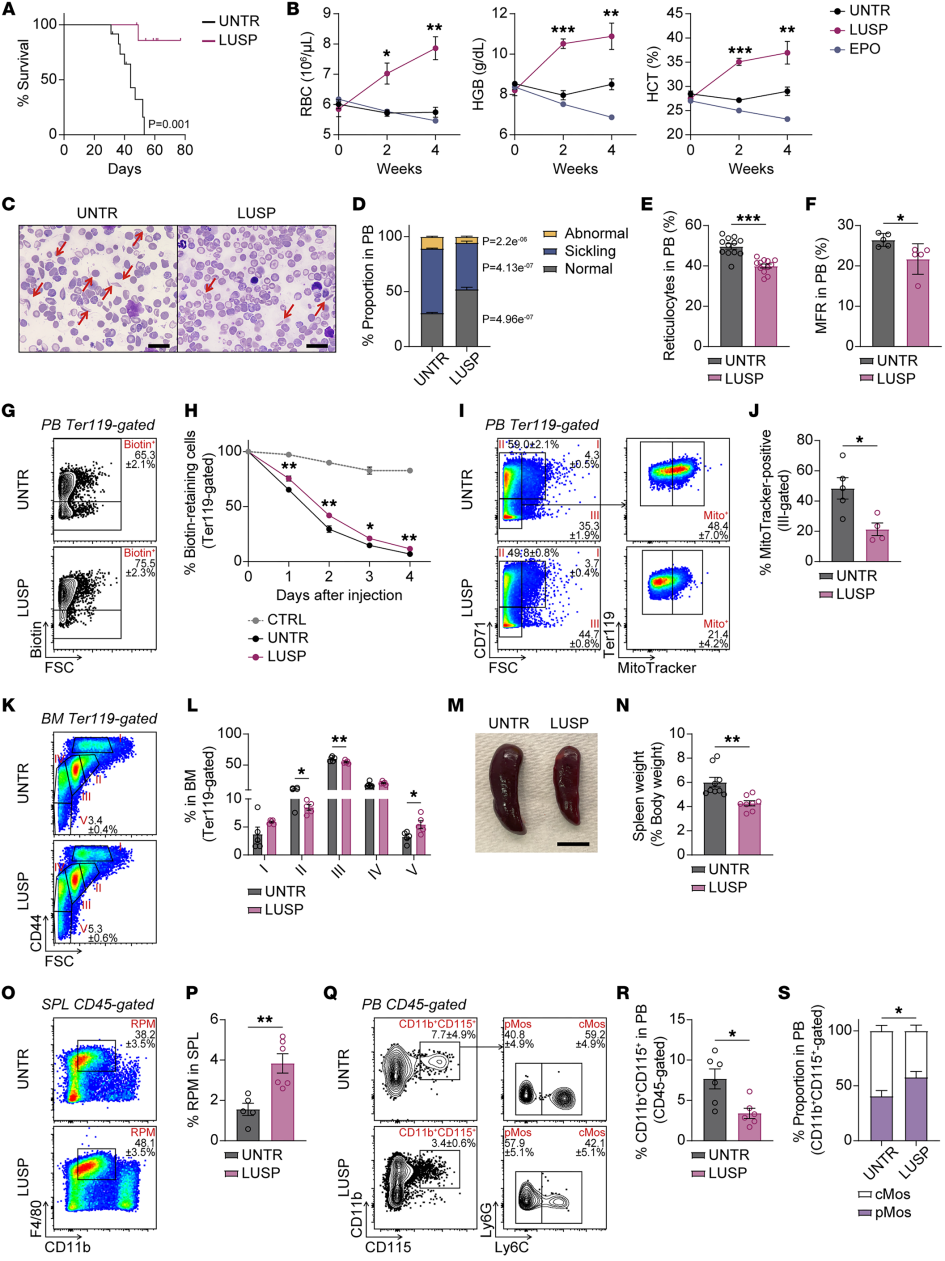
Luspatercept alleviates anemia in the Townes SCD mouse model. (**A**) Kaplan-Meier survival curve of untreated (UNTR) and luspatercept-treated (LUSP) SCD mice (*n* = 9–12). (**B**) Average PB RBC, hemoglobin (HGB), and hematocrit (HCT) levels in untreated and treated mice (*n* = 4–7). (**C**) Giemsa staining of PB smears. Arrows mark sickling cells. Scale bars: 20 μm. (**D**) Proportion (%) of normal, sickling, and abnormal cells (*n* = 4–6 independent experiments). (**E**) Average percentage reticulocytes (*n* = 12) in PB. (**F**) Average percentage MFR reticulocytes (*n* = 5). (**G**) Flow plots of biotin-retaining cells in PB. (**H**) Quantification of percentage biotin-retaining cells on days 0 to 4 (*n* = 9–13). (**I**) Flow plots of PB erythroid cells and their mitochondrial content. (**J**) Quantification of percentage MitoTracker-positive cells (*n* = 4–5). (**K**) Flow plots of BM erythroid maturation (I, proerythroblasts; II, basophilic; III, polychromatic; IV, orthochromatic and reticulocytes; V, RBCs). (**L**) Quantification of percentage BM erythroid progenitors (*n* = 6). (**M**) Representative image of spleens. Scale bar: 0.5 cm. (**N**) Spleen weight as percentage body weight (*n* = 8–9). (**O**) Flow plots of splenic red pulp macrophages (RPMs). (**P**) Quantification of percentage RPMs (*n* = 5–6). (**Q**) Flow plots of CD11b^+^CD115^+^ cells, cMos, and pMos. (**R**) Quantification of percentage CD11b^+^CD115^+^ cells (*n* = 6). (**S**) Ratio of cMos versus pMos (*n* = 6). *P < 0.05; **P < 0.01; ***P < 0.001 by 2-way ANOVA with Tukey’s post hoc test for **B** and **H**, and 2-tailed Student’s *t* test for all others.

## Version 1 02/02/2026

Electronic publication
