# Supplementary material for: Luspatercept ameliorates disease phenotype and complications in the Townes mouse model of sickle cell disease
Source: J Clin Invest. 2026 Feb 2;136(3):e197706. doi: 10.1172/JCI197706 (PMC12867147; doi:10.1172/JCI197706)
Supplement: Supplemental data [file jci-136-197706-s318.pdf]

Supplemental Methods

Sex as a biological variable

In this study, sex was not considered as a biological variable. Both male and female mice were used.

Mice

The Townes sickle cell mice, purchased from The Jackson Laboratory (strain #027265), were bred and maintained in the cleanroom at UT Health San Antonio. The genotypes hα/hα::β^A/β^S and hα/hα::β^S/β^S were used as control and experimental mice, respectively. Of note, all experiments were conducted on young mice (from 4 weeks of age) to account for the gradual replacement of fetal hemoglobin (HbF) to undetectable levels with sickle hemoglobin (HbS) by 4 weeks, impacting overall mice survival.

In vivo drug treatment

SCD mice were i.p. injected with 30 mg/kg luspatercept (MCE; HY-P99720) twice a week (i.e., Tuesday and Friday) for at least 3 weeks. Likewise, SCD mice were treated with 1660 U/kg of erythropoietin (EPO) (MyBioSource; MBS650094). Blood was collected using heparinized capillaries, and CBC readings were measured on the Element HT5+ (HEKSA).

RBC lifespan and mitochondrial content of PB cells

Mice were i.v. injected with 1 mg EZ-Link™ Sulfo-NHS-Biotin (Thermo Fisher Scientific; 21217) diluted in 100 µL of PBS. After 2 hours, blood was collected using heparinized capillaries for day (D)0 analysis. Cells were first incubated with MitoTracker Green for 15 minutes at 37°C, followed by staining with streptavidin-BV421 (for biotin detection) and erythroid progenitor markers, CD44 and Ter119 for 30 minutes on ice.

Flow cytometry

Bone marrow (BM) cells were flushed with FACS buffer (2% FBS in PBS), centrifuged at 300 g for 5 minutes at 4°C and red blood cells (RBCs) were lysed with RBC lysis buffer (BioLegend; 420301). Spleens were mashed onto a mesh filter using a syringe plunger. For HSPC analysis, cells were stained with biotin-conjugated lineage antibodies (IL-7Rα, Gr-1, CD3e, Ter119, CD11b, CD4, B220, Nk1.1, CD4) for 20 minutes at 4°C, followed by fluorochrome-conjugated HSPC antibodies (cKit, Sca-1, CD48, CD150) for 30 minutes on ice. For all other stainings, cells were first incubated in 10% FBS in PBS for 10 minutes to block Fc receptors. The following antibodies were used for the detection of

erythroid lineages (Ter119, CD44, CD71), red pulp macrophages (F4/80, CD11b, Ter119, CD163, TIM4), classical/patrolling monocytes (CD11b, CD115, Ly6C, Ly6G) and MEPs (Lineage, cKit, Sca-1, CD16/32, CD34). Cells were resuspended in FACS buffer with 2 µg/mL Hoechst 33342 (Thermo Fisher Scientific; 62249) to distinguish live/dead cells and acquired on the BD FACSCelesta Cell Analyzer. Fetal globin was stained according to a previously described protocol by Yu *et al* (1). Data was analyzed using FlowJo (BD; version 10.8.1). All antibodies can be found in Suppl. Table 1.

Fluorescence immunohistochemistry and quantification

Long bones were fixed overnight in 4% PFA at 4°C, rinsed, cryoprotected with 30% sucrose in PBS, embedded in O.C.T Compound (Sakura Finetek; 4583), flash frozen and both sides were cut longitudinally using a cryostat to expose the BM surface for antibody penetration. Sections were blocked with blocking buffer (10% donkey serum, 0.02% Triton-X and 0.1% BSA in PBS) overnight at 4°C, stained with primary antibodies in blocking buffer overnight at 4°C, washed with PBST and stained with secondary antibodies overnight at 4°C. Samples were cleared using RapiClear (SUNJin Lab; RC152001) and imaged on the Leica STELLARIS confocal. Images were quantified as averages from 3 separate locations and a minimum of 50 cells per plane from 3 *z*-planes, and biological replicates of $n = 3$ bones per group. All antibodies can be found in Suppl. Table 1.

Histology

Cytospin slide preparation: BM cells were resuspended in 250 µL of FACS buffer and centrifuged at 500 rpm for 5 minutes using the Epredia Cytospin 4 (Epredia; A78300003). Slides were air-dried for 2 hours minimum, fixed in methanol for 10 minutes, and stained. For Giemsa May-Grünwald staining, slides were processed according to the instructions provided by the manufacturer (Sigma; GS-10). All images were captured using the BZ-X series Keyence microscope.

Statistics

Significance was determined using the Student's two-tailed t-test for two comparisons and two-way ANOVA with Tukey's post hoc test for multiple comparisons. A P value < 0.05 was considered significant. A minimum of three independent experiments was performed for each analysis. Data are shown as mean ± SEM. Figures were generated using the Prism and Adobe Illustrator software.

Study approval

All animal studies were conducted according to an approved Institutional Animal Care and Use

Committee (IACUC) protocol and federal regulations at UT Health San Antonio.

Data availability

Values for all data points in graphs are reported in the Supporting Data Values file.

Acknowledgements

This study was supported by the NIH R01 Grants (CA248019 and CA266256 to GH), the DoD Grant (W81XWH2110148 to GH), and the P30 Cancer Center Support Grant (CA054174 to GH). This work is the result of NIH funding, in whole or in part, and is subject to the NIH Public Access Policy. Through acceptance of this federal funding, the NIH has been given the right to make the work publicly available in PubMed Central.

Author contributions

M.S. designed the research, performed experiments, analyzed results, generated figures, and wrote the manuscript; **T.L.** performed experiments and acquired data; **M.P.** performed experiments and acquired data; **Z.W.** performed experiments and analyzed results; **J.B.** performed experiments; **J.H.** provided material/resources and interpreted data; **J.Z.X.** interpreted data and edited the manuscript; **G.H.** designed the research, wrote the manuscript, and supervised the study.

References

1. Yu L, et al. Identification of novel γ-globin inducers among all potential erythroid druggable targets. *Blood Adv*. 2022;6(11):3280-3285.

Supplemental Table 1

Antibodies	Clone	Company	Cat. #	Experiment
Biotin anti-mouse CD127 (IL-7Ra)	A7R34	eBioscience	13-1271-85	Flow (lineage)
Biotin rat anti-mouse Gr-1	RB6-8C5	BD Biosciences	553125	Flow (lineage)
Biotin hamster anti-mouse CD3e	145-2C11	BD Biosciences	553060	Flow (lineage)
Biotin rat anti-mouse Ter119	TER-119	BD Biosciences	553672	Flow (lineage)
Biotin rat anti-mouse CD11b	M1/70	BD Biosciences	553309	Flow (Lineage)
Biotin rat anti-mouse CD45R/B220	RA3-6B2	BD Biosciences	553086	Flow (Lineage)
Biotin anti-mouse NK1.1	PK136	BioLegend	108703	Flow (Lineage)
Biotin rat anti-mouse CD4	RM4-5	BD Biosciences	553045	Flow (Lineage)
Biotin rat anti-mouse CD8a	53-6.7	BD Biosciences	553029	Flow (Lineage)
APC anti-mouse CD117	2B8	BioLegend	105812	Flow (HSPC)
PerCP/Cy5.5 rat anti-mouse Sca-1	D7	BioLegend	108124	Flow (HSPC)
PE/Cy7 rat anti-mouse CD150	TC15-12F12.2	BioLegend	115913	Flow (HSPC)
APC/Cy7 anti-mouse CD48	HM48-1	BioLegend	103432	Flow (HSPC)
BV510 anti-mouse CD45	30-F11	BioLegend	103138	Flow (RPM)
FITC anti-mouse Ter119	TER-119	eBioscience	11-5921-85	Flow (RPM)
PerCP/Cy5.5 rat anti-mouse TIM4	RMT4-54	BioLegend	130020	Flow (RPM)
PE/Cy7 anti-mouse CD11b	M1/70	BioLegend	101216	Flow (RPM)
APC anti-mouse F4/80	BM8	BioLegend	123115	Flow (RPM)
APC/Cy7 anti-mouse CD163	S15049I	BioLegend	155324	Flow (RPM)
MitoTracker Green	-	Invitrogen	M7514	Flow (Mitochondria)
BV510 rat anti-mouse CD11b	M1/70	BD Biosciences	562950	Flow (cMos, pMos)
PE anti-mouse CD115	AFS98	BioLegend	135505	Flow (cMos, pMos)
PerCP/Cy5.5 anti-mouse Ly6G	1A8	BioLegend	127616	Flow (cMos, pMos)
PE/Cy7 anti-mouse Ly6C	HK1.4	BioLegend	128018	Flow (cMos, pMos)
APC/Cy7 rat anti-mouse CD45	30-F11	BD Biosciences	557659	Flow (cMos, pMos)
PE rat anti-mouse CD34	RAM34	BD Pharmingen	551387	Flow (MEP)
PE/Cy7 anti-mouse CD16/32	93	Invitrogen	25-0161-82	Flow (MEP)
PE rat anti-mouse CD71	C2	BD Biosciences	553267	Flow (EP)
APC anti-mouse fetal hemoglobin	HBF-1	Invitrogen	MHFH05	Flow (HbF)
BV785 anti-mouse/human CD44	IM7	BioLegend	103041	Flow (EP)

AF647 anti-mouse Ter119	TER-119	BioLegend	116218	Flow (EP)/IHC
Anti-rat endomucin	V.7C7	Santa Cruz Biotechnology	sc-65495	IHC
Cy3-donkey anti-rat IgG secondary	-	Jackson ImmunoResearch	712-165-153	IHC
**Reagents**		**Company**	**Cat. #**	**Experiment**
RapiClear 1.52		SUNJIN LAB	RC152001	IHC
Hoechst 33342		Invitrogen	H3570	Flow (live/dead)
DAPI		Invitrogen	D1306	Flow/IHC
Luspatercept		MCE	HY-P99720	*In vivo* drug treatment
Erythropoietin		MyBioSource	MBS650094	*In vivo* drug treatment
EZ-Link™ Sulfo-NHS-Biotin		ThermoFisher Scientific	21217	RBC lifespan

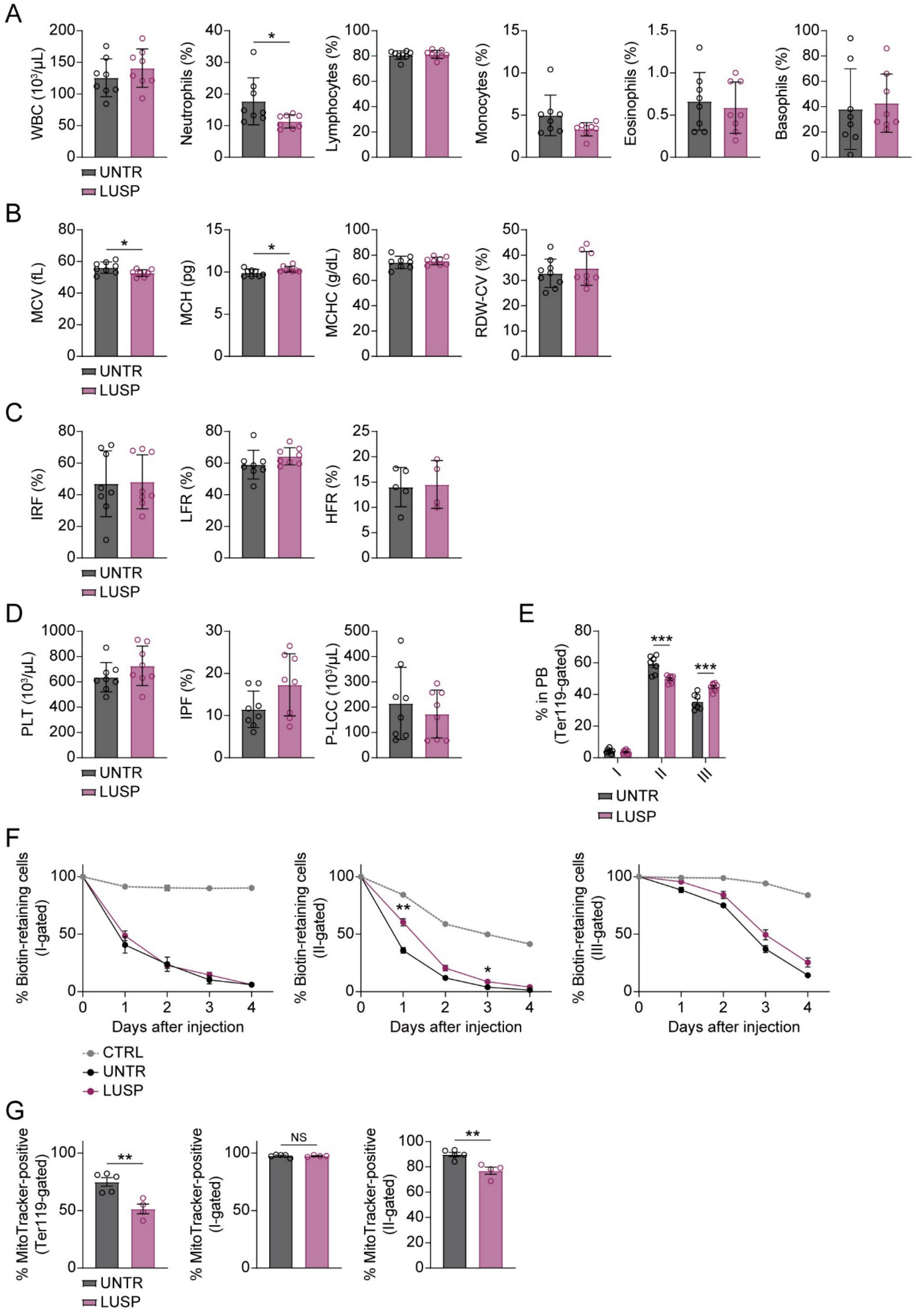

Supplemental Figure 1. Luspatercept acts on RBCs in the Townes mouse model.

(A) WBC parameters of untreated and luspatercept-treated peripheral blood (PB) from SCD mice. (B) RBC parameters of untreated and luspatercept-treated PB: mean corpuscular volume (MCV; fL), mean corpuscular hemoglobin (MCH; pg), mean corpuscular hemoglobin content (MCHC; g/dL), red blood cell distribution width-coefficient of variation (RDW-CV; %). (C) Reticulocyte parameters of untreated and luspatercept-treated PB: immature reticulocyte fraction (IRF; %), low fluorescent ratio (LFR; %), high fluorescent ratio (HFR; %). Reticulocytes can be classified based on their RNA content, which reflects their level of maturation (immature with HFR to MFR and mature with LFR). (D) Platelet parameters of untreated and luspatercept-treated PB: platelet (PLT; 10^3/μL), immature platelet fraction (IPF; %), platelet-large cell count (P-LCC; 10^3/μL) (*n* = 8 mice per group for A-D). (E) Quantification of % erythroid cells in PB subsets I-, II-, and III-gated cells (*n* = 7-8 mice per group). (F) Quantification of % biotin-retention in PB subsets I- (left), II- (middle), and III-gated (right) cells from pulse-chase day (D)0 to 4 (*n* = 3-5 mice per group). (G) Quantification of % MitoTracker-positive Ter119- (left), and subsets I- (middle) and II-gated (right) cells in PB of untreated and luspatercept-treated SCD mice (*n* = 4-5 mice per group). *, $P < 0.05$; **, $P < 0.01$; ***, $P < 0.001$; NS, not significant by two-way ANOVA with Tukey's post hoc test for 1F and Student's t-test for all others. Data are shown as mean ± SEM.

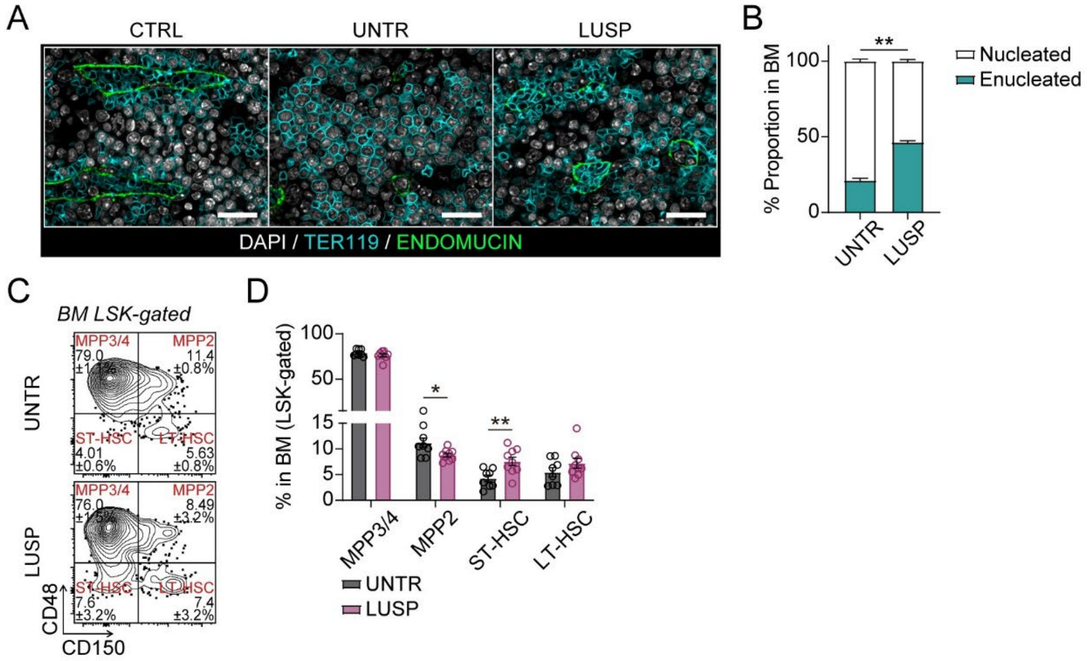

Supplemental Figure 2. Luspatercept boosts mature RBCs in the BM and rescues stem cells. (A) Bone marrow (BM) imaging of control, untreated, and lusaptercept-treated SCD mice showing a reduction in nucleated and immature Ter119-positive erythroid cells (white = DAPI; cyan = Ter119; green = Endomucin). Scale, 20 µm. (B) Ratio of nucleated to enucleated cells in untreated and luspatercept-treated BM from SCD mice (average of 50 cells per image from 3 *z*-planes at 3 separate locations per mouse, *n* = 3 mice per group). (C) Representative flow plots of hematopoietic stem and progenitor cells (HSPCs) in untreated and luspatercept-treated BM from SCD mice. (D) Quantification of % LSK-gated BM HSPCs in C (*n* = 8-9 mice per group). *, P < 0.05; **, P < 0.01. Student's t-test unless otherwise specified. Data are shown as mean ± SEM.

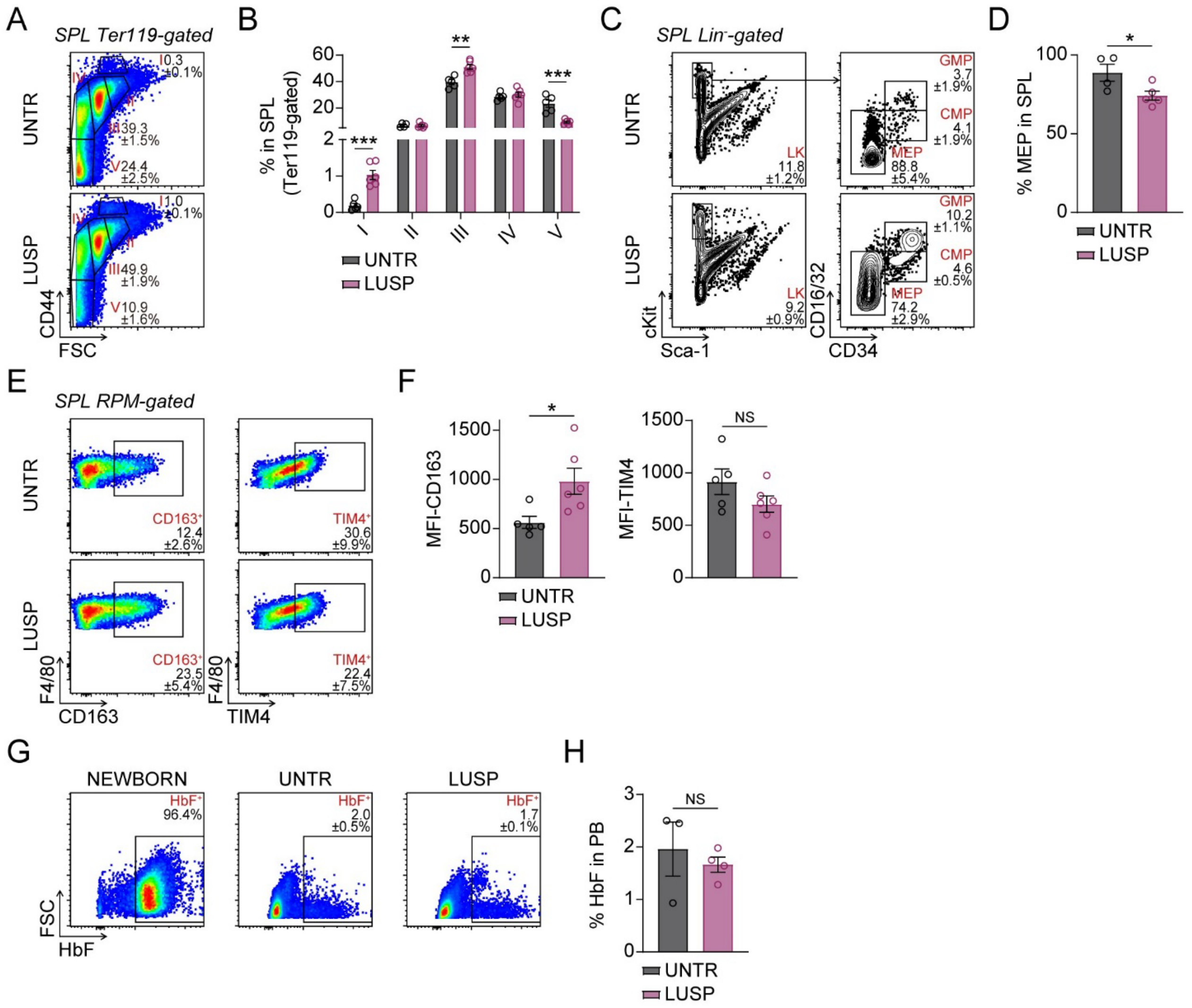

Supplemental Figure 3. Luspatercept reduces dependence on EMH.

(A) Representative flow plots of erythroid maturation in Ter119-gated spleen cells of untreated and luspatercept-treated SCD mice. (B) Quantification of % erythroid progenitors in A (*n* = 6-7 mice per group). (C) Gating scheme for granulocyte-monocyte progenitors (GMPs), common myeloid progenitors (CMPs), and megakaryocyte-erythroid progenitors (MEPs). (D) Quantification of % MEPs in untreated and luspatercept-treated spleen cells (*n* = 4-5 mice per group). (E) Representative flow plots of CD163 (left) and TIM4 (right) expression in RPM-gated untreated and luspatercept-treated spleen cells. (F) Mean fluorescent intensity (MFI) of RPM markers CD163 (left) and TIM4 (right) (*n* = 5-6 mice per group). (G) Representative flow plots of fetal hemoglobin (HbF) staining in newborn, untreated, and luspatercept-treated PB. (H) Quantification of % HbF in PB (*n* = 3-4 mice per group). *, P < 0.05; **, P < 0.01; ***, P < 0.001; NS, not significant. Student's t-test unless otherwise specified. Data are shown as mean ± SEM.
